# Salt Bridges Regulate Both Dimer Formation and Monomeric Flexibility in HdeB and May Have a Role in Periplasmic Chaperone Function

**DOI:** 10.1016/j.jmb.2011.11.026

**Published:** 2012-01-20

**Authors:** Wenjian Wang, Tim Rasmussen, Amanda J. Harding, Nuala A. Booth, Ian R. Booth, James H. Naismith

**Affiliations:** 1 Biomedical Sciences Research Complex, University of St Andrews, St Andrews, Fife KY16 9ST, UK; 2 Institute of Medical Sciences, University of Aberdeen, Foresterhill, Aberdeen AB25 2ZD, UK; 3 Laboratory of Department of Surgery, The First Affiliated Hospital, Sun Yat-sen University, 58 Zhongshan RoadⅡ, Guangzhou 510080, China

**Keywords:** crystal structure, fluorescence measurements, hydrophobic residues, acid response, pH titration

## Abstract

*Escherichia coli* and Gram-negative bacteria that live in the human gut must be able to tolerate rapid and large changes in environmental pH. Low pH irreversibly denatures and precipitates many bacterial proteins. While cytoplasmic proteins are well buffered against such swings, periplasmic proteins are not. Instead, it appears that some bacteria utilize chaperone proteins that stabilize periplasmic proteins, preventing their precipitation. Two highly expressed and related proteins, HdeA and HdeB, have been identified as acid-activated chaperones. The structure of HdeA is known and a mechanism for activation has been proposed. In this model, dimeric HdeA dissociates at low pH, and the exposed dimeric interface binds exposed hydrophobic surfaces of acid-denatured proteins, preventing their irreversible aggregation. We now report the structure and biophysical characterization of the HdeB protein. The monomer of HdeB shares a similar structure with HdeA, but its dimeric interface is different in composition and spatial location. We have used fluorescence to study the behavior of HdeB as pH is lowered, and like HdeA, it dissociates to monomers. We have identified one of the key intersubunit interactions that controls pH-induced monomerization. Our analysis identifies a structural interaction within the HdeB monomer that is disrupted as pH is lowered, leading to enhanced structural flexibility.

## Graphical Abstract

**Figure:**
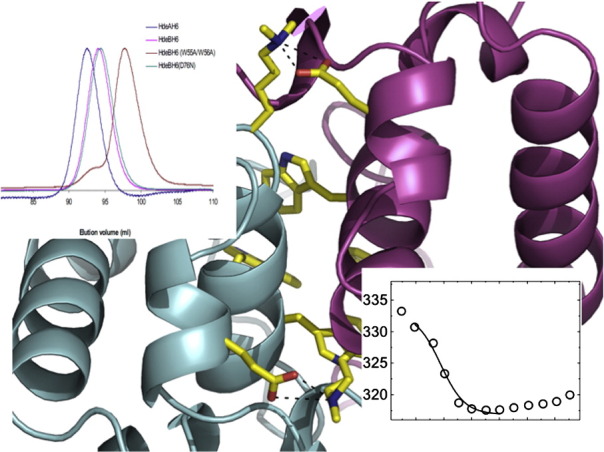

**Research Highlights:**

► HdeB, an acid-activated chaperone, forms a dimer at neutral pH. ► The dimer reversibly dissociates at pH 3.2. ► The dissociation is mainly regulated by an intersubunit salt bridge. ► An internal salt bridge is broken at low pH, possibly enhancing structural flexibility.

## Introduction

Bacterial cells that passage through the stomach en route to colonization of the gut must survive extremely low external pH of around 2.^1^ At this pH, many critical proteins will unfold and aggregate with lethal consequences for the organism. Bacteria protect their cytoplasmic components by buffering their internal pH and thus preventing such lethal changes in the external environment.^2,3^ However, the periplasm, due to weak buffering and the presence of porins in the outer membrane, does suffer large drops in pH in response to changes in extracellular environment. Two proteins, HdeA and HdeB, were discovered as abundant periplasmic proteins in stationary-phase *Escherichia coli* cells, and genetic studies implicated them in survival of extremely acid pH.^3–6^ Subsequently, the *hdeAB* operon has been shown to be subject to the complex regulation that is associated with many proteins that are required for survival of acid stress.^6–9^ Unusually for stress proteins, which are normally widespread among bacterial species, the distribution of HdeA and HdeB is extremely narrow, being restricted to selected members of the Proteobacteriaciae (Table S1).

HdeA and HdeB have been shown, *in vitro*, to have acid-activated chaperone functions; that is, by binding to acid-denatured proteins, they prevent these other proteins from irreversible aggregation and precipitation.^4,10^ At a molecular level, the proteins sequester the exposed hydrophobic surface of target proteins. At neutral pH, HdeA, which is the more intensively studied protein of the pair, exists as a homodimer that has no ability to bind to other proteins. The structure of HdeA has been solved to 2 Å resolution at neutral pH and reveals that the monomer is primarily α-helical and forms a dimer with an extensive and hydrophobic interface.^4,11^ Upon incubation at acid pH (∼ pH 2), HdeA is activated and forms monomers. It is this newly exposed dimer interface of HdeA that binds to hydrophobic faces of the denatured proteins, preventing their aggregation. This poses a challenge on how the specific dimer interface seen in HdeA can recognize such a wide range of substrates. Here, we report the crystal structure of HdeB at pH 4.5 and biophysical characterization of HdeB at both acid and neutral pH. Like HdeA, HdeB is dimeric at neutral pH but forms monomers at low pH (∼ pH 3.5). By monitoring the intrinsic fluorescence of HdeB as pH changes, we have identified additional changes in structure that accompany acid-induced monomer formation.

## Results

### HdeB is a dimer

**Fig. 1:**
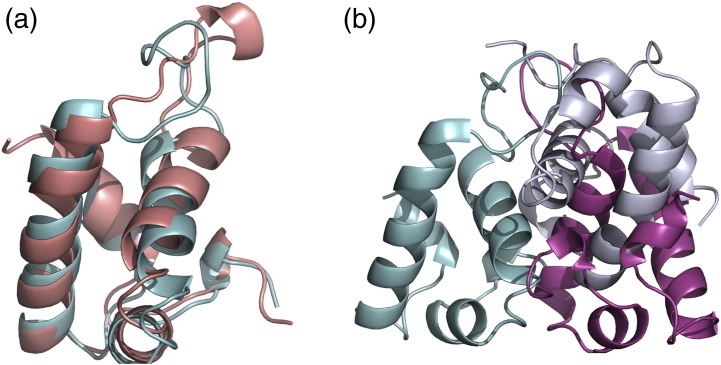
The structure of HdeB. (a) The structure of HdeB (cyan) superimposed with HdeA (orange).^11^ The principal difference is in the loop (residues 64 to 72 in HdeB) connecting two helices. (b) Dimer of HdeB with monomers colored cyan (subunit A) and maroon (subunit B). The HdeB dimer is very different from the HdeA dimer.^11^ The B subunit of the HdeA is shown in gray; its position is based on superposition of subunit A of HdeA (not shown) onto subunit A of HdeB (cyan).

The asymmetric unit of the crystal contains four protein monomers with residues A30 to A102 ordered; six C-terminal residues and the His-tag are disordered. The N-terminal 29-residue periplasmic export sequence is predicted by SignalP to be removed during processing.^12^ Thus, the structure represents the whole of the mature protein in experimental electron density. The monomeric structure is composed of two 12-residue helices (α2 and α3), three short (4 or 5 residues) helices (α1, α4, and α5), and interconnecting loops (Fig. 1a). Despite low sequence identity between HdeA and HdeB (13%), the monomeric structure of HdeB is very close to that observed for HdeA^4,11^ (1dj8 and 1bg8) as had been predicted by threading analysis.^4^ In total, 61 C^α^ atoms superimpose with an RMSD of 1.75 Å. The major difference occurs in the long loop that connects the two large helices (residues 64 to 72 in HdeB). The superposition of the structures shows that the two loops generate an almost ‘figure of eight’ arrangement (Fig. 1a). The difficulty in tracing the HdeB loop structure from molecular replacement models had hindered our refinement.

**Fig. 2:**
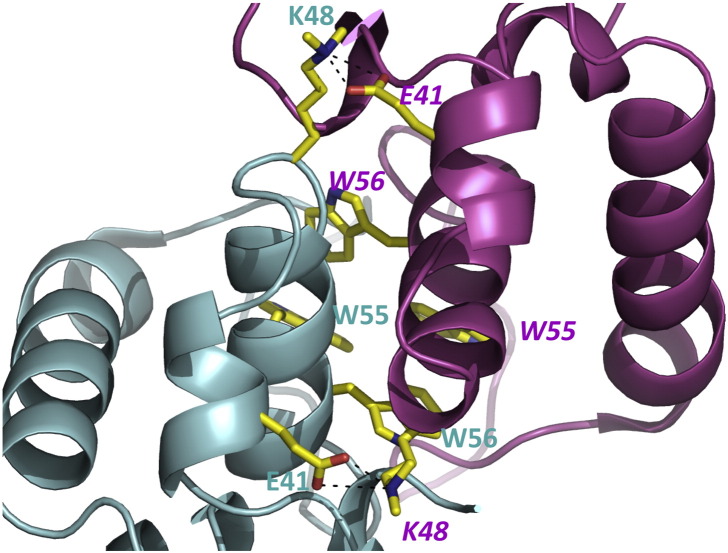
Salt bridges and tryptophan cluster at the HdeB dimer interface. The dimer interface has a cluster of four tryptophan residues (W55 and W56 from each subunit). The two intermolecular salt bridges (K48 and E41) are shown. Residues from subunit A are labeled in cyan; those from subunit B are labeled in maroon.

Analysis with PISA^13^ reveals that the four monomers in the asymmetric unit are arranged as two identical dimers of HdeB. The dimers themselves make no significant contacts with each other. The dimer is identified as stable by the program PISA^13^ and the dimer interface buries in total 2400 Å^2^ of surface area, much of it involving hydrophobic residues (Fig. 1b). There are six main-chain intersubunit hydrogen bonds (akin to a β-sheet interaction) formed between the loop from each monomer that connects the two long helices. The α2 helix from each monomer makes extensive intersubunit interactions, as does the N-terminus of the mature protein. Two tryptophan residues (W55 and W56) and a tyrosine residue (Y64) from each monomer come together to make a cluster of aromatic residues at the C-terminal end of α2 (Fig. 2). This hydrophobic cluster is a striking feature of the HdeB dimer. We constructed the double mutant (W55A, W56A) to support our structural model and indeed the double mutant is folded but is found only as a monomer at pH 7 (Fig. S1). For K48, which is at the interface and methylated, the Nz atom makes a salt contact with the side chain of E41 of the other subunit (Fig. 2). The methyl groups point towards the solvent, suggesting that the methyl groups do not perturb the dimer interface.

The HdeA dimer buries 2200 Å^2^ of surface area, which also predominantly involves hydrophobic residues, but interestingly, it has a quite different arrangement from that seen for HdeB. The two α2 helices in the HdeA dimer are almost parallel, whereas they are almost perpendicular in the HdeB dimer (Fig. 1b). In HdeA, the side chains of the α2 helix on one monomer fill a groove on the surface of the other monomer. This groove is absent in HdeB and is instead filled by side chains from within the monomer (including W55 and W56). The different dimer arrangements seen for HdeA and HdeB are mutually exclusive since, unless there are significant conformational changes, the arrangements would cause extensive van der Waals clashes. Further, the residues engaged in each dimer interface are not conserved between the proteins. We conclude that the different arrangement of the HdeB dimer observed in the crystal is real rather than artifactual. Analogous to the behavior observed for HdeA, gel-filtration analysis shows that the dimer of HdeB can be fully dissociated into monomers at pH 2.5 (Fig. S1) and that this dissociation is fully reversible by increasing the pH. CD spectra at different pH values showed that HdeB retained its overall structure through the range in which it dissociates but that significant structural changes may occur in the monomer at very low pH (< pH 3).

### Spectroscopic characterization of the pH-dependent dissociation of the HdeB dimer

**Fig. 3:**
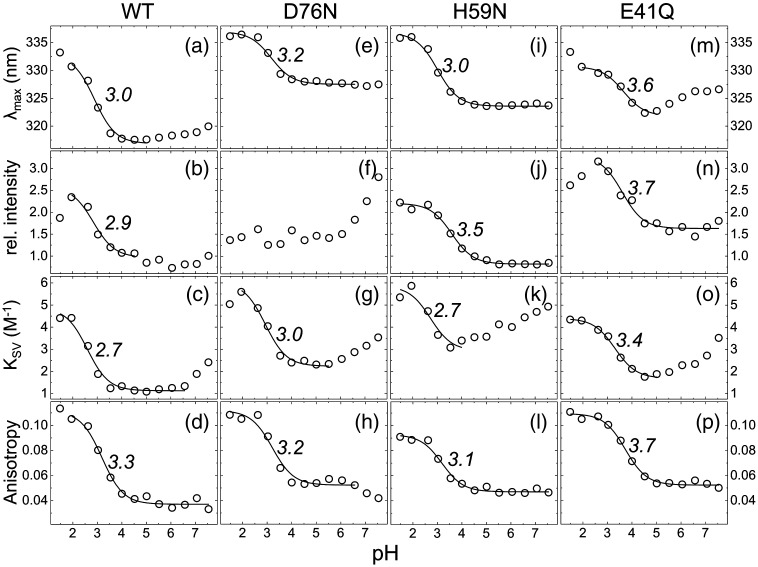
pH dependence of HdeB tryptophan fluorescence. Four different fluorescence properties over a pH range of 1.5 to 7.5 are shown: maximum of the emission peak, λ_max_ (first row); relative intensity (second row); Stern–Volmer constant of quenching with acrylamide, *K*_SV_ (third row); and steady-state fluorescence anisotropy (fourth row). These properties are displayed for HdeB WT (first column) and the mutants D76N (second column), H59N (third column), and E41Q (fourth column). The data (circles) were fitted with Eq. (3) (lines), and the obtained apparent p*K*_a_ values are given.

The position of the only tryptophan residues (W55 and W56) at the dimer interface (Fig. S2) suggested that they could be used as intrinsic probe to monitor changes in the dimeric state of HdeB by fluorescence spectroscopy. Emission spectra, steady-state fluorescence anisotropies, and quenching curves were recorded in the range from pH 1.5 to 7.5 for the purified HdeB-His_6_ WT (Fig. 3a–d). Significant changes in the emission properties and in quenching were observed when the pH was varied. The emission maximum moved to longer wavelengths (red shift) below around pH 3, indicating an increase in polarity of the immediate environment of the tryptophan residues, as would be expected from dimer-to-monomer transition and consistent with gel-filtration results (Fig. S1 and Ref. 10). Quenching experiments follow a similar trend; below pH 3, much higher Stern–Volmer acrylamide quenching constants were obtained, indicating that the tryptophan residues become exposed to bulk water (Fig. 3c). Fitting of these data to Eq. (3) revealed an effective p*K*_a_ ≈ 3 for dissociation of the dimer. Similar pH dependence was obtained for fluorescence anisotropy. These changes are likewise fully reversible by increasing the pH (Fig. S2). The data convincingly identify pH-dependent dissociation of the dimer.

### Dimer dissociation; role of the intersubunit salt bridge E41 and K48

**Fig. 4:**
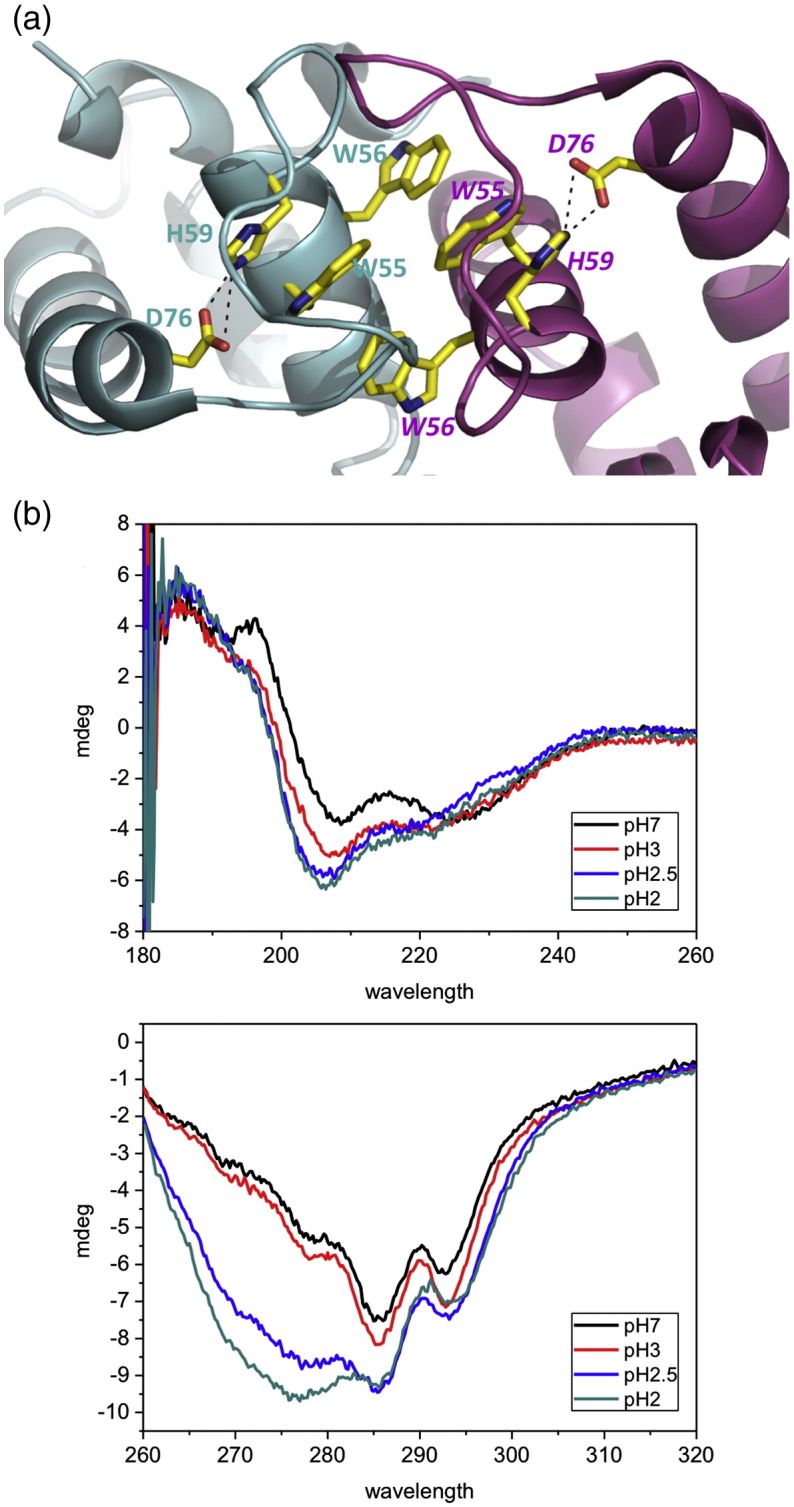
The internal salt bridges within HdeB. (a) In each monomer, a salt bridge between D76 and H59 is present. The residues are colored as in Fig. 2. (b) CD spectra of HdeB WT at different pH values: (top) far-UV CD and (bottom) near-UV CD. The CD spectrum of HdeB WT shows pH-dependent changes indicating changes in the secondary and tertiary structures, respectively.

**Fig. 5:**
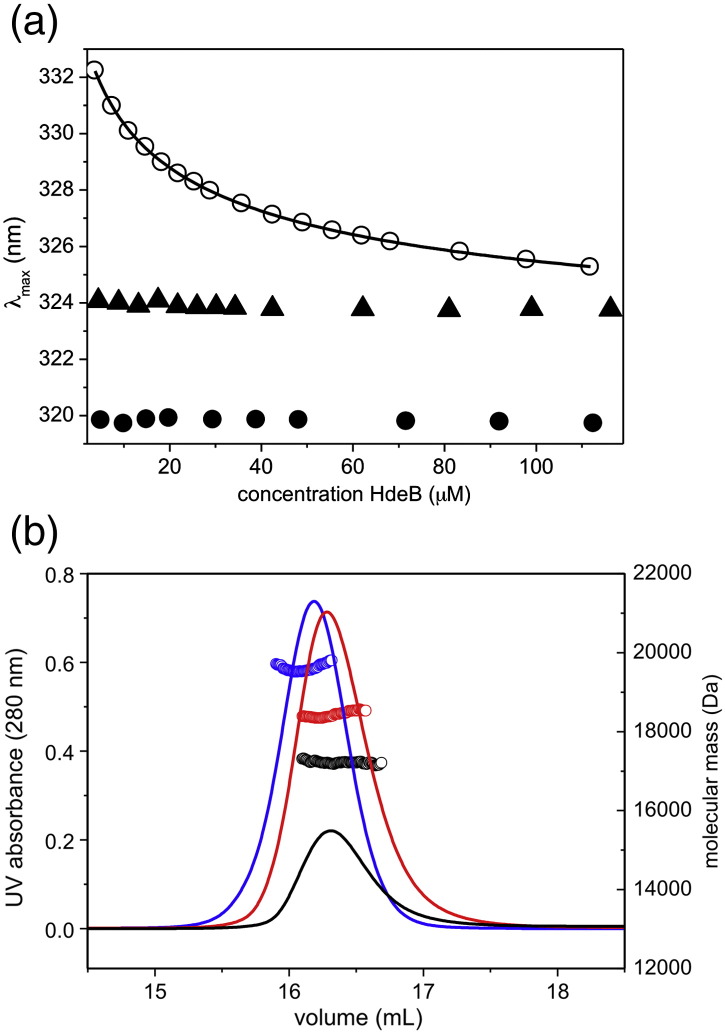
Concentration-dependent dissociation of HdeB E41Q. (a) The spectral position of the emission maximum is shown in relation to HdeB concentration for WT (filled circles) and the mutants H59N (triangles) and E41Q (open circles). A fit of the E41Q data to Eq. (9) is shown as a line. Concentrations are given as HdeB monomers. (b) Size-exclusion profiles of purified HdeB are given as absorbance at 280 nm (lines). Forty nanomoles  of HdeB WT (blue) and E41Q (red) was injected. In addition, a smaller amount (10 nmol) of E41Q (black) was applied. Molecular masses over each profile were calculated from static light-scattering data (circles).

The tryptophan residues are clearly essential for dimer formation, but it is difficult to explain how they could control pH sensitivity. The crystal structure shows an intersubunit salt bridge at the dimer interface of HdeB: E41 from one subunit with K48 from the other (Fig. 2) and internal salt bridge within each monomer D76–H59 (Fig. 4a). We created the E41Q mutant to break the intermolecular salt bridge and determined the fluorescence properties of the purified mutant protein. Although the E41Q mutant still undergoes a pH-dependent dissociation at 50 μM, with consequent increase in λ_max_ and quenching, it does so with an apparent p*K*_a_  ∼ 0.5 pH units higher than that of wild type (Fig. 3m, o, and p). Gel-filtration data suggested a mixture of dimer and monomer, even at neutral pH. We therefore investigated the concentration dependence of the dimeric state. The emission maximum wavelength was observed to shift as a function of the protein concentration at pH 7.5 (Fig. 5a; 1–100 μM range). Fitting of these data yields λ_M_ = 334.4 ± 0.4 nm and λ_D_ = 320.9 ± 0.3 nm at low (monomer) and high (dimer) concentrations, respectively. These values agree well with λ_max_ values found for wild-type monomer (acid pH) and dimer (neutral pH), respectively. Light scattering revealed a slight reduction in apparent average mass with 20 kDa *versus* 17 kDa for wild type and E41Q, respectively (Fig. 5b). A simple dissociation curve yields a *K*_d_ = 34 ± 10 μM for the E41Q dimer; no such dissociation is observed for wild type, indicating a *K*_d_ below 1 μM. We therefore conclude that this salt bridge is indeed critical for the stability of the dimer and that its loss destabilizes the dimer by at least 2 orders of magnitude. The fact that the destabilized dimer still undergoes pH-dependent dissociation suggests that other salt bridges may be present. However, none are evident in the native structure, and consequently, these may only form in the mutant. The most likely candidate we predict would be E42, which is close to E41 in the tertiary structure and, in the mutant, could partially substitute for E41, making a salt bridge to K48.

### Influence of pH upon monomer structure; internal salt bridge

We noted that for the native protein, the fluorescence intensity changes in response to pH, with the effect titrating at pH 2.9. Although this could be attributed to dissociation, we investigated further to see whether there were additional pH-induced changes around the tryptophan residues. Asp76 makes a polar contact with the indole nitrogen of Trp55 and a salt bridge with His59, which in turn stacks against the indole of W55 (Fig. 4a). The hydrogen bond between D76 and H59 links α3 and α2 helices together. We reasoned that the D76–H59 salt bridge may be disrupted at low pH, and this in turn could perturb tryptophan fluorescence. The D67N mutant exhibited the same monomer–dimer pH transition as wild type (as judged by gel filtration), and analysis of fluorescence data indicated that the monomer-to-dimer transition occurred with a p*K*_a_ identical with that of the wild-type protein (Fig. 3e, g, and h). These data are consistent with the internal salt bridge having no role in dimer formation, which is also consistent with the structure of HdeB where the His59–Asp76 salt bridge is remote from the dimer interface. However, the D76N protein exhibited profound differences in the spectroscopic properties of the Trp residues; notably, the emission intensity was constant in the pH 1.5–6 range (in contrast to wild type, which titrates with p*K*_a_ = 2.9, Fig. 3f) and the emission maximum wavelength was red shifted (Fig. 3e). Since this titration is not possible for D76N, we attribute the changes in fluorescence intensity in the wild-type protein arising from protonation of D76, which changes the charge in the direct environment of the tryptophan,^14,15^ rather than protonation of either D76 or H59, causing pH-dependent dissociation of the HdeB dimer. Although the p*K*_a_ of 2.9 for D76 is below the 3.5 normally found for aspartic residues in proteins,^16^ the involvement of Asp76 in a salt bridge with His59 would be expected to lower its p*K*_a_. Protonation of histidine to change structure and regulate function is extremely common. A recent example is *E. coli* DegQ protease, which degrades misfolded proteins and undergoes changes in activity and oligomeric state in the pH range associated with His protonation, although no formal evidence for this mechanism has been presented.^17^ The system in HdeB is different, in that it is the protonation of Asp76 involved in a salt bridge with H59 that mediates a subtle structural change. The D76N data also reveal an increase in intensity at higher pH (> 6.5) much greater than that seen in the wild type. We ascribe this to deprotonation of H59 at higher pH values, which in its protonated (charged) form would also be expected to quench tryptophan.^18^ The difference between mutant and wild type is explained by a salt bridge with Asp76 that, when present, would raise the p*K*_a_ of His59. To confirm this interpretation, we created the H59N mutant, which shows a single titratable p*K*_a_ of 3.5, consistent with a simple D76 protonation event, but no major modification of the dimer–monomer transition (Fig. 3i, j, and l).

At pH 6, both D67N and H59N exhibit a substantial red shift in the emission maximum and a corresponding offset of the quenching curves (Fig. 3e and i) relative to native, suggesting increased access to water. The salt bridge between D76 and H59 thus appears to be important in maintaining the local structural environment around the tryptophan residues, probably due to linking α3 and α2. Since the Asp76–His59 salt bridge breaks at about the same pH as observed for monomer formation, we predicted that the structure of the protein monomer would likewise change at about the same point the dimer breaks. This hypothesis is supported by CD spectroscopy, where changes in secondary and tertiary structure are observed at low pH (Fig. 4b). We suggest that this indicates that dimer dissociation is accompanied by conformational change within the monomer.

## Discussion

Chaperones frequently exert their action by providing hydrophobic surfaces that sequester hydrophobic groups that are exposed during either folding or unfolding of other proteins.^19^ The HdeAB proteins fulfill this role for periplasmic proteins in some Gram-negative bacteria, and the observation that their hydrophobic face is buried until the proteins are exposed to low pH has led to their being implicated in stabilizing proteins that are destabilized at low pH.^4,10^ Paradoxically, the HdeAB system enjoys a very narrow distribution in the microbial kingdom from which we infer that these chaperones do not provide a universal panacea to acid stress-induced protein denaturation. The crystal structures show that HdeA and HdeB have distinct hydrophobic interfaces that are exposed at different low pH values, perhaps indicating different targets. Although each protein has been demonstrated to have chaperone activity with ‘artificial’ (i.e., non-periplasmic or non-*E. coli*) proteins, their substrates in their host organism remain unclear.^4,10^ For HdeA, partial unfolding in acid is proposed to be required to adapt to different substrates.^20^ Recent work has suggested that the HdeA protein has a very narrow substrate range, including other chaperones and protein-folding proteins.^21^ However, the substrate profile of HdeB remains unknown. We have determined the structure of HdeB and the mechanism of its acid sensing, identifying two critical salt bridges that are broken upon exposure to low pH. This leads us to speculate that HdeB could work by means of a two-step mechanism. Firstly, the inter-monomer salt bridge, when broken, destabilizes the HdeB dimer significantly (at least 100-fold). Secondly, the internal salt bridge within the HdeB monomer is broken, and this increases structural flexibility, particularly at the newly exposed interface. Enhancing the flexibility of the newly exposed interface is potentially a molecular mechanism by which HdeB may be able to deform to chaperone different target protein structures.^10^

## Materials and Methods

### Structural biology

Hdeb-His_6_ was expressed and purified after cloning of the *hdeAB* genes from *E. coli* into pTrc99A (Pharmacia) using a PCR product generated with primers Hdeb-His_6_-F and Hdeb-His_6_-R (Supplementary Data) that integrate appropriate restrictions sites, to create plasmid pTrcHdeABHis_6_. The primers were designed such that a His_6_-tag was fused in-frame to the carboxy-terminus of HdeB. The insert in the plasmid was sequenced on both strands to ensure retention of the *E. coli* coding sequence and the in-frame His-tag. Expression in strain MG1655Δ*hdeAB* was induced by growth with 1 mM IPTG and cell extracts prepared after 18 h of growth (i.e., after entry into stationary phase). The expressed protein was shown to complement the HdeB deficiency in acid survival experiments (data not shown). Purification of Hdeb-His_6_ from the extract used Ni resin, protein was eluted using 50–100 mM imidazole, and the eluted material was subjected to further purification by gel filtration. The identity and integrity of the purified protein were verified by peptide mass spectrometry.

After gel filtration, HdeB was concentrated to 5, 10, and 15 mg ml^− 1^. The protein was screened for crystallization against a range of conditions from commercial kits in standard use at the Scottish Structural Proteomic Facility.^22^ The protein crystallized under a number of conditions, but we failed to obtain any diffraction data for the crystals. The protein was modified by reductive methylation following a published protocol.^23^ After methylation, gel filtration was rerun, and a small shift was observed in elution (to a higher mass), but the peak remained sharp and consistent with a dimer; crystal trials were repeated. After methylation, the protein yielded crystals with sharp edges with protein at 15 mg ml^− 1^ in cryo-47 (Emerald BioSystems): 50% (v/v) polyethylene glycol 400, 0.1 M acetate, pH 4.5, and 0.2 M Li_2_SO_4_. The crystals were taken directly from the drop and cryo-cooled prior to data collection. Data were recorded to 1.5 Å at the European Synchrotron Radiation Facility and processed with MOSFLM.^24^ Molecular replacement using HdeA failed to give a solution that we were able to refine with confidence. Selenomethionine-variant protein was produced using the method of Doublie.^25^ The variant was treated identically to the native and yielded crystals under the same conditions. Data were collected at the European Synchrotron Radiation Facility. Selenium sites were identified, and the structure was phased using the SHELX suite of programs.^26^ The model was built with ARP/wARP,^27^ refined with REFMAC5,^28^ and adjusted by Coot.^29^ TLS groups were defined using the TLSD server^30^ and used throughout refinement. Individual atomic thermal parameters were refined isotropically. Crystallographic statistics are given in Table 1. There is unambiguous electron density for doubly methylated lysine residues (K48, K88, and K93) and clear density for some modification of others (K35, K65, K89, and K99), with only K82 apparently unmodified in the crystal. In two monomers, there is electron suggestive of methylated tyrosine (Y64 in chain A); however, we could not confirm this by mass spectrometry, and the additional density could be due to different rotamers.

### Gel-filtration and circular dichroism analysis

Hdeb-His_6_ was concentrated to approximately 0.3 mg ml^− 1^ in 10 mM sodium phosphate or 10 mM citrate buffer depending on the required pH. A series of CD spectra were recorded from pH 2 to pH 7. The near-UV region (260–310 nm) shows that the protein undergoes a profound conformational change below pH 3. This change can be reversed by increasing the pH back to 7.

### Fluorescence spectroscopy

Purified samples of Hdeb-His_6_ were diluted in McIlvaine buffer to a final concentration of 50 μM. McIlvaine buffer is a mixture of 0.1 M citric acid and 0.2 M Na_2_HPO_4_ at different ratios to buffer at a pH between 2.6 and 7.^31^ For more acid solutions, a  10-mM KCl solution was adjusted with hydrochloric acid. Fluorescence spectra were recorded with an FLS920 spectrometer (Edinburgh Instruments) as described earlier.^32^ Briefly, 200 μl of sample was excited at 295 nm with an excitation path of 10 mm and an emission path of 4 mm while both slits were set to 2 nm. The temperature was kept at 20 °C. The spectra were fitted to a skewed Gaussian:^33^

(1) $$I=I_{\text{max}}\exp\left(-\left(\text{ln}2\right){\left[\text{ln}\left(1+2b\left(\lambda -\lambda_{\max}\right)/w_{\lambda }\right)/b\right]}^{2}\right)+a$$

with fluorescence intensity *I* at wavelength λ and *I*_max_ at λ_max_, skew parameter of *b*, peak width at half-height of *w*_λ_, and baseline offset of *a*. Intensities were normalized to the emission intensity of HdeB-His_6_ WT at pH 7.5.

Quenching experiments were performed by titrating a  1-M stock solution of acrylamide to the sample up to a concentration of 0.2 M. The intensities at 340 nm were fitted to the Stern–Volmer equation:

(2) $$I_{0}/I=1+K_{\text{SV}}\left[\text{Q}\right]$$

*I* and *I*_0_ represent the fluorescence intensities with and without quencher, respectively; *K*_SV_ is the quenching constant; and [Q] is the concentration of acrylamide.

Parameters obtained from fitting the spectra (*I*_max_, λ_max_), *K*_SV_ from the quenching experiments, and the fluorescence anisotropy were fitted to Eq. (3) to obtain an apparent p*K*_a_ value:

(3) $$P=\left(P_{\text{A}}\left[{\text{H}}^{+}\right]+P_{\text{B}}K_{\text{a}}\right)/\left(K_{\text{a}}+\left[{\text{H}}^{+}\right]\right)$$

where *P* is one of the spectroscopic parameters at a specific pH; *P*_A_ and *P*_B_ are the parameters for the acidic and basic form, respectively; [H^+^] is the proton concentration; and *K*_a_ is the equilibrium constant. As discussed above, the mutant D76N showed that changes in the intensities were independent of changes in the other parameters reflecting different processes. Therefore, *P*_A_ and *P*_B_ for λ_max_, *K*_SV_, and anisotropy directly indicated concentrations of species and required no correction for intensities. Data analysis and fitting were performed using the software Origin 8.0 (OriginLab). pH-dependent measurements were performed at least in duplicate, and mean values for the apparent p*K*_a_ values are given.

The dissociation constant *K*_d_ for HdeB E41Q was determined from the concentration-dependent change in peak position λ_max_ at pH 7.5.

(4) M+M⇔D

(5) $$K_{\text{d}}={\left[\text{M}\right]}^{2}/\left[\text{D}\right]$$

*K*_d_ can be expressed dependent on the degree of dissociation α = [M]/*c*, where *c* is the total concentration of HdeB given as monomers^34^ [Eq. (6)]

(6) $$K_{\text{d}}=2\alpha^{2}c/\left(1-\alpha \right)$$

Equation (6) solved for α results in:

(7) $$\alpha =\left({\left(8cK_{\text{d}}+{K_{\text{d}}}^{\text{2}}\right)}^{1/2}-K_{\text{d}}\right)/4c$$

pH-dependent measurements on HdeB D76N suggest that the quantum yields for the monomer and the dimer are similar (see Results). Therefore, α can be obtained from measurements of the peak position λ_max_ according to Eq. (8), which also contains the peak positions for the pure dimer and monomer, λ_D_ and λ_M_:

(8) $$\alpha =\left(\lambda_{\text{D}}-\lambda_{\max}\right)/\left(\lambda_{\text{D}}-\lambda_{\text{M}}\right)$$

Substitution of Eq. (8) in Eq. (7) results in:

(9) $$\lambda_{\max}=\lambda_{\text{D}}-\left(\lambda_{\text{D}}-\lambda_{\text{M}}\right)\left({\left(8cK_{\text{d}}+{K_{\text{d}}}^{\text{2}}\right)}^{1/2}-K_{\text{d}}\right)/4c$$

λ_D_ and λ_M_ were difficult to estimate from experiments directly but fittings of Eq. (9) to λ_max_ were sensitive to these parameters so that they were varied together with *K*_d_ using Origin 8.0. Experiments were performed in triplicate and mean and standard deviation are given.

### Static light scattering

A Wyatt miniDAWN TREOS multiangle light-scattering instrument, together with a Wyatt Optilab T-rEX refractometer, was used, coupled online to an AKTA Purifier (GE Healthcare) pump system. An analytical  25-ml Superdex 200 size-exclusion column (GE Healthcare) was equilibrated with 50 mM phosphate buffer, pH 7.5 (containing 150 mM NaCl), and purified HdeB-His_6_ WT and E41Q samples were injected. Molecular masses were calculated using the ASTRA software (Wyatt) according to the manufacturer. Bovine serum albumin (Thermo Fisher) and rabbit aldolase (Sigma) were used as controls. Refractive index increments were calculated on the basis of the amino acid sequence using the software Sedfit,^35^ and extinction coefficients were calculated using the ExPASy online suite.

### Accession numbers

Coordinates and structure factors have been deposited in the Protein Data Bank with accession number 2xuv.

## Figures and Tables

**Table 1:** Crystallographic data

	HdeB (methylated)	Se-Met HdeB (methylated)
Source	Diamond IO2	Diamond IO2
Wavelength (Å)	0.9796	0.9775
Space group	*C*2	*C*2
Unit cell dimensions		
*a* (Å)	100.9	100.2
*b* (Å)	86.5	86.1
*c* (Å)	48.5	48.3
α (°)	90	90
β (°)	112.5	112.6
γ (°)	90	90
Wilson *B*-factor (Å^2^)	19	
Resolution (Å)	33–1.5 (1.54–1.5)	46–2.2 (2.22–2.20)
Unique reflections	58,562 (4060)	19,209 (544)
Mosaicity (°)	0.24	0.68
Anomalous correlation	—	0.72 (0.66)
Completeness (%)	95.3 (94.5)	99.8 (100)
Multiplicity	3.8 (3.8)	5.4 (5.5)
Mean *I*/σ	24 (2.7)	19 (9.7)
*R*_merge_ (%)	3.8 (21.1)	5.6 (11.0)
*Refinement*		
*R*-factor/*R*_free_ (%)	18.1/19.5 (19.3/22.8)	
RMSD bonds (Å)/angles (°)	0.01/1.35	
MolProbity score/centile	1.31/95	
Number of atoms	2707	
PDB code	2xuv	

Se-Met, selenomethionine.
