# Supplementary material for: Salt Bridges Regulate Both Dimer Formation and Monomeric Flexibility in HdeB and May Have a Role in Periplasmic Chaperone Function
Source: J Mol Biol. 2012 Jan 20;415(3):538–46. doi: 10.1016/j.jmb.2011.11.026 (PMC3299563; doi:10.1016/j.jmb.2011.11.026)
Supplement: Supplementary file 1 — Supplementary materials [file mmc1.pdf]

Salt bridges regulate both dimer formation and monomeric flexibility in HdeB and may have role in periplasmic chaperone function

Wenjiang Wang[1, 2, 3], Tim Rasmussen[2,4], Amanda J Harding[2,4], Nuala A. Booth[4], Ian. R Booth[4*] & James H. Naismith[1*]

[1] Biomedical Sciences Research Complex, The University, St Andrews, UK KY16 9ST

[4] Institute of Medical Sciences, Foresterhill, The University, Aberdeen UK AB25 2ZD

[2]equal contribution;

[*]communicating authors

Ian R. Booth i.rbooth@abdn.ac.uk Phone 44-(0)1224 437396

James H. Naismith naismith@st-and.ac.uk Phone 44-(0)1334-463792

[3] Current address: Laboratory of Department of Surgery, The First Affiliated Hospital, The University,Sun Yat-Sen, Guangzhou, China 510080

Supporting material

Table S1

Figure S1

Figure S2

Table S1. Distribution of HdeA and HdeB homologues among microbial genera.

Genus	HdeA	HdeB
Shigella	3e-49	8e-76
Escherichia*	7e-48	2e-68
Enterobacter	6e-41	6e-26
Yersinia	**	2e-24
Salmonella	**	2e-24
Edwardsiella	**	5e-24
Klebsiella	**	7e-19
Providencia	2e-23	2e-17
Citrobacter	**	9e-09
Achromobacter	5e-23	**
Laribacter	1e-21	**
Brucella	4e-19	**
Bordatella	9e-18	**
Thiocapsa	5e-13	**
Methylocystis	4e-11	**
Methylobacter	5e-06	**

Whole genome sequences were probed with either HdeA or HdeB proteins (including the signal sequence). Coverage for the alignments was >65% and most variation was in the signal sequence region, indicating that the functional mature protein was moderately to highly conserved.

**Escherichia* species other than *E. coli* variants. **not detected at the level of genome reporting of orfs.

Figure S1

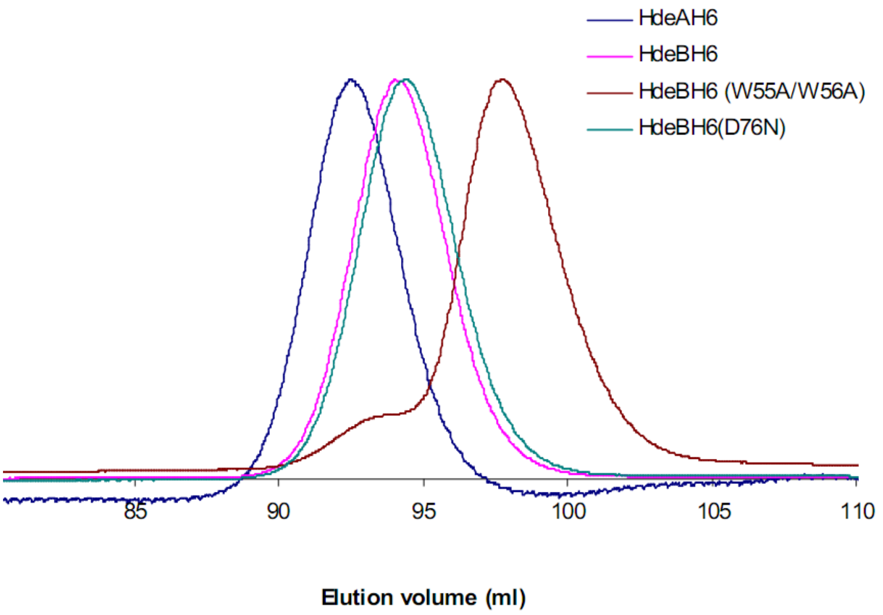

Figure S1: Size exclusion chromatography of HdeA and HdeB at pH 7: Profiles for HdeA WT (blue), HdeB WT (pink), and the HdeB mutants W55A/W56A (brown) and D76N (cyan) are shown.

Figure S2

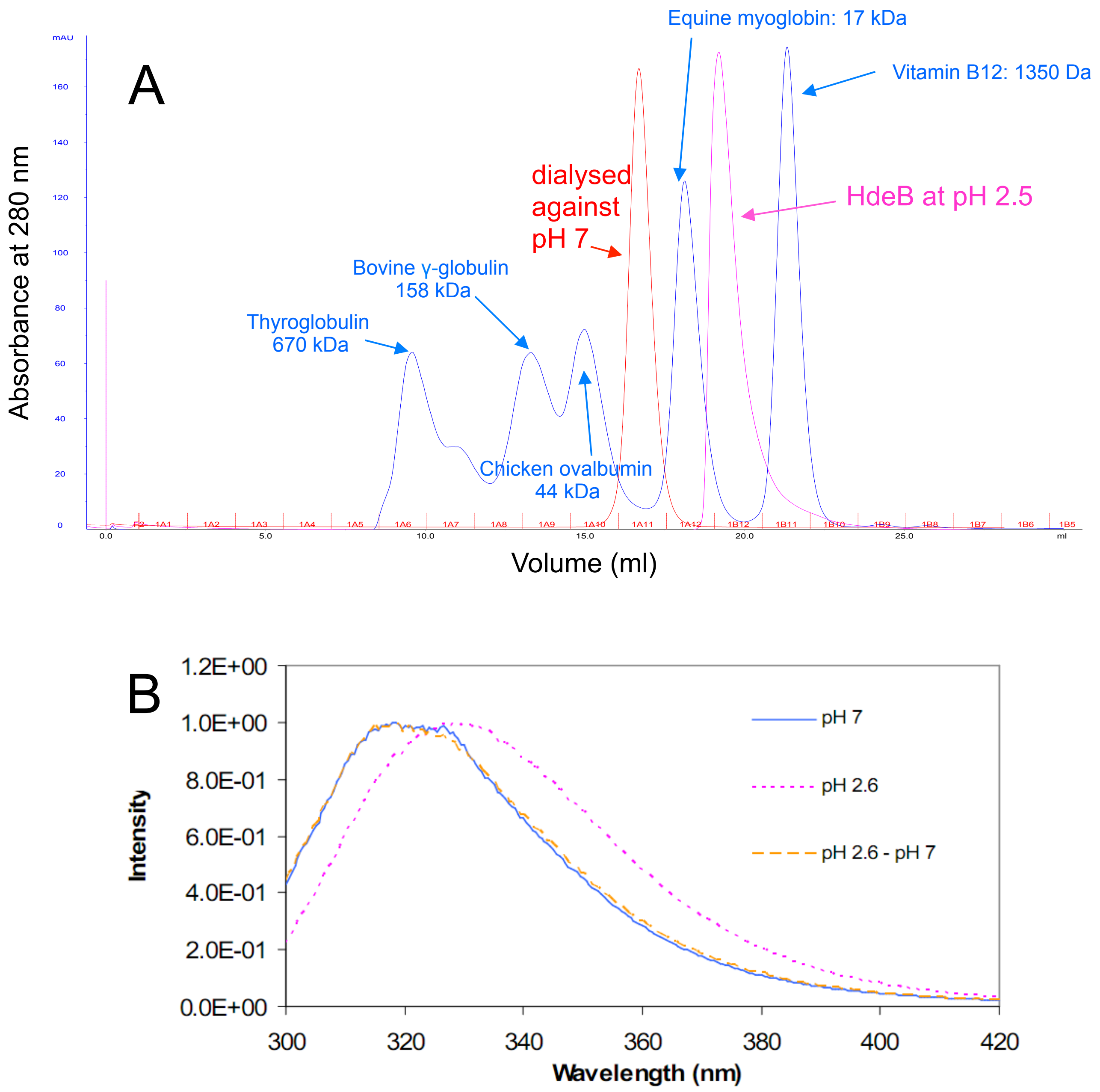

Figure S2: Reversibility of dissociation of HdeB after neutralisation: (A) pH-dependence of size exclusion chromatography of HdeB. Profiles are shown for acid-treated HdeB WT at pH 2.5 (pink). The second half of the acid-treated sample was dialysis against buffer at pH 7.0 and run at this pH (red). The position of the dialysed sample corresponds to the position of HdeB that has not been acid-treated. Standards with their molecular masses are shown in blue. (B) Emission spectrum of HdeB WT at pH 7 (blue), at pH2.6 (pink), and after neutralisation from pH 2.6 to 7.0 (yellow).
